# Relationship assessment of microbial community and cometabolic consumption of 2-chlorophenol

**DOI:** 10.1007/s00253-025-13403-7

**Published:** 2025-01-24

**Authors:** Miguel Martínez-Jardines, Omar Oltehua-López, Sergio Martínez-Hernández, Anne-Claire Texier, Flor de María Cuervo-López

**Affiliations:** 1 Department of Biotechnology, Universidad Autónoma Metropolitana-Iztapalapa, Av. Ferrocarril San Rafael Atlixco 186, Col. Leyes de Reforma 1A Sección, Iztapalapa, CDMX Mexico City, Mexico; 2 Institute of Biotechnology and Applied Ecology, Universidad Veracruzana, Av. de Las Culturas Veracruzanas 101, 91090 Xalapa, Veracruz, Mexico

**Keywords:** 2-Chlorophenol, Cometabolic consumption, Correlation analysis, Nitrifying process, Population dynamics, Sequencing batch reactors

## Abstract

**Abstract:**

The relationship of microbial community and cometabolic consumption of 2-chlorophenol (2-CP) in a nitrifying sequencing batch reactor (SBR) was studied. The assessment of the population dynamics of the nitrifying sludge during the cometabolic 2-CP consumption with increasing ammonium (NH_4_^+^) concentrations in the SBR showed the presence of 39 different species of which 10 were always present in all cycles. Fifty-five percent of the species found were grouped as Proteobacteria (45% as β-proteobacteria and 10% as γ-proteobacteria class), 30% as Acidobacteria, and 15% as Deinococcus-Thermus phyla. NH_4_^+^ and cometabolic 2-CP consumption could be related to the presence and permanence of ammonium-oxidizing bacteria (AOB) species and heterotrophic bacteria, while the complete nitrification to the presence of nitrite-oxidizing bacteria (NOB) species. A correlation analysis showed that the complete and stable nitrifying performance (NH_4_^+^ consumption efficiencies (ENH_4_^+^-N) > 99% and nitrate production yields (YNO_3_^−^-N) between 0.93 and 0.99), as well as the increase in specific rates (ammonium (qNH_4_^+^-N) and 2-CP (q2-CP-C) consumption and nitrate production (qNO_3_^−^-N)), was associated with the homogeneity of the bacterial community (*J* index = 0.99). The increase in the proportion of individuals of AOB species such as *Nitrosomonas oligotropha* and *Nitrosomonas marina* was associated with the increase in qNH_4_^+^-N (*r* ≥ 0.69) and q2-CP-C (*r* ≥ 0.64) and, therefore, with the 2-CP cometabolic consumption in the SBR. Finally, the increase in the proportion of individuals of heterotrophic species such as *Dokdonella ginsengisoli*, *Deinococcus peraridilitoris*, *Truepera radiovictrix*, and *Stenotrophobacter terrae* was associated with the increase in q2-CP-C (*r* ≥ 0.59).

**Key points:**

• *Thirty-nine bacterial species were identified in the nitrifying sludge population of the SBR.*

• *β-Proteobacteria and Acidobacteria were the prevalent (85%) bacterial groups.*

• *AOB and heterotrophic bacteria participate in NH*_*4*_^*+*^* and cometabolic 2-CP consumption.*

**Graphical abstract:**

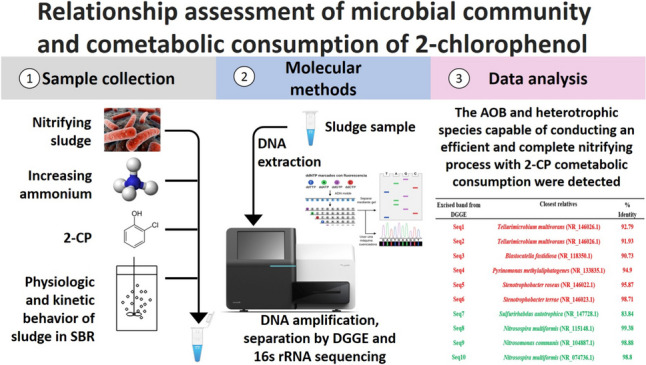

**Supplementary Information:**

The online version contains supplementary material available at 10.1007/s00253-025-13403-7.

## Introduction

Nitrification, a two-stage aerobic respiratory process, has been extensively investigated as the first step of nitrogen removal from wastewater. At the first stage, ammonium (NH_4_^+^) is sequentially oxidized to hydroxylamine (NH_2_OH) by the ammonium monooxygenase (AMO) enzyme and later to nitrite (NO_2_^−^) by the activity of the enzyme hydroxylamine oxidoreductase (HAO) of the ammonium-oxidizing bacteria (AOB). In the second stage, NO_2_^−^ is oxidized to nitrate (NO_3_^−^) by nitrite-oxidizing bacteria (NOB) through the action of the nitrite oxidoreductase (NOR) enzyme (Van Kessel et al. 2015; Jaramillo et al. 2018).

In recent years, the nitrification contribution in the micropollutants, recalcitrant or emerging compounds elimination has been emphasized and associated with the AOB because of the AMO enzyme participation in the cometabolic consumption of these compounds (Yu et al. 2018; Kumwimba and Meng 2019). Cometabolism has been defined as the ability of microorganisms to transform a substrate that is not used as growth support in the mandatory presence of a growth substrate or an easily transformable or biodegradable compound under aerobic or anaerobic conditions (Nzila 2013; Jesus et al. 2016). Microbial cometabolism is a fundamental part of the biological elimination of several toxic and recalcitrant compounds that by themselves could not be eliminated; moreover, in some cases, high rates of degradation and low accumulation of intermediates can be achieved (Jesus et al. 2016).

There are works showing that under nitrifying conditions and high NH_4_^+^ loading rates, greater biodegradation rates and consumption efficiencies of organic and emerging compounds have been obtained; besides, the AMO enzyme has been suggested to play an important role in the cometabolic biodegradation of various organic pollutants (Tran et al. 2013; Martínez-Jardines et al. 2018; Trejo-Castillo et al. 2021). Nevertheless, in several of the studies found in the literature, response variables that evaluate the physiologic and/or kinetic behavior and population dynamics of nitrifying sludge are not included. Thus, it remains in doubt whether the cometabolic consumption was carried out in association with the nitrifying process, as well as the impact on the microbial community and the stability of the nitrifying performance.

It has been reported that the feeding of NH_4_^+^ concentrations greater than 100 mg/L NH_4_^+^-N throughout the operation cycles in nitrifying sequencing batch reactor (SBR) can be advantageous to the growth and selection of some species of AOB such as *Nitrosomonas* in the sludge (Terada et al. 2013). However, no statistical analysis was performed to corroborate the relationship among NH_4_^+^ concentration, operating cycles, and the growth of nitrifying species. In this sense, the composition and/or changes of the microbial community in a nitrifying consortium constantly exposed to recalcitrant, toxic, or emerging compounds and to high concentrations of NH_4_^+^ could also be associated with the development and achievement of the cometabolic process under nitrifying conditions. In this regard, in most of the works where molecular biology studies were carried out under nitrifying conditions, information on changes in microbial communities by modifying environmental conditions (Wang et al. 2010) or by increasing or decreasing substrate (NH_4_^+^) and/or organic loads is provided (Silva et al. 2014; Zielińska et al. 2014). However, during the cometabolic consumption of recalcitrant compounds, the relationship among kinetic and physiologic response variables and the population dynamics supported by statistical analysis has not been explored.

A better understanding of the microbial behavior and functioning of the microbial consortia implicated in the biological processes under certain operating conditions would allow a better grasp on how cometabolic processes occur, as well as the behavior and function of the microorganisms present in biological reactors. Particularly, more research is needed to understand the population dynamics of nitrifying sludge during cometabolic consumption of 2-chlorophenol (2-CP). Knowledge of the relationship among the physiologic and kinetic response of the nitrifying sludge during the cometabolic consumption of 2-CP, the participation of the AMO enzyme in the consumption of NH_4_^+^ and the recalcitrant compounds, and population dynamics of nitrifying sludge would be helpful for establishing more stable and efficient treatment systems, with higher working capacity and longer operating times during the elimination of inhibitory and/or recalcitrant compounds from wastewater.

Hence, the aim of this study was to evaluate the relationship among the population dynamics of a microbial consortium with the nitrifying respiratory process and the cometabolic consumption of 2-CP in SBR. The evaluation throughout operation cycles of the physiologic and kinetic behavior of the nitrifying sludge and 2-CP cometabolic consumption was directed by using consumption efficiencies, NO_3_^−^ production yields, and specific rates of NH_4_^+^ and 2-CP consumption and NO_3_^−^ generation. The changes in the bacterial community were monitored by using denaturing gradient gel electrophoresis (DGGE) and sequencing of DGGE fragments, for calculating ecological indices and the proportion of individuals throughout the operating cycles in SBR. Finally, the association of population dynamics with the physiologic and kinetic response of the nitrifying sludge was evaluated by the Pearson correlation statistical analysis.

## Materials and methods

### Nitrifying kinetic assays in batch and SBR systems: cometabolic 2-CP consumption by AMO enzyme

The physiologic and kinetic behavior of the nitrifying sludge, as well as the cometabolic 2-CP consumption assays conducted in both batch and SBR systems, has been previously evaluated and described in Martínez-Jardines et al. (2018). A short description of the experimentation carried out is presented. Two nitrifying sequencing batch reactors (SBR1 and SBR2) of 1.2 L of operation volume were used. The nitrifying process and cometabolic consumption of 2-CP (60 mg/L 2-CP-C) supplemented with different initial concentrations of NH_4_^+^ (100–500 mg/L NH_4_^**+**^**-**N) were conducted in the SBRs during 13 operation cycles. Operation cycles consisted of the following periods: filling = 0.02 days; reaction time = 14–21 days; settle = 0.02 days, and draw = 0.083 days. The SBRs were inoculated with nitrifying sludge (354.7 ± 9.9 mg/L of microbial protein as biomass), remained sealed to avoid 2-CP losses, and were intermittently aerated twice a day with O_2_ (99% purity) for maintaining a dissolved oxygen (DO) concentration of 4.1 ± 1.7 mg/L. The initial pH medium was set at 8.1 ± 1.3. The SBRs were maintained at room temperature and stirred at 250 rpm.

Nitrification and the participation of the AMO enzyme in the presence of 40 mg/L allylthiourea (ATU) as a specific inhibitor of AMO and 5 mg/L 2-CP-C were evaluated in serological bottles with the nitrifying sludge used as inoculum for the SBR and with the sludge obtained at the end of the 13 operating cycles. The physiologic and kinetic behavior of nitrification and 2-CP consumption was determined by consumption efficiencies of NH_4_^+^ (ENH_4_^+^-N) and 2-CP (E2-CP-C), NO_3_^−^ production yields (YNO_3_^−^-N), biomass production yields (YBM), and specific rates of NH_4_^+^ (qNH_4_^+^-N) or 2-CP (q2-CP-C) consumption and NO_3_^−^ production (qNO_3_^−^-N) as response variables.

### Molecular methods

Sludge samples were withdrawn from each SBR at the end of each operating cycle (13 cycles). The samples were centrifuged at 5000 rpm for 10 min, and the pellets were frozen at − 20 °C until DNA extraction was conducted. DNA was extracted using the UltraClean™ Soil DNA Isolation Kit (MO BIO Laboratories, Carlsbad, CA, USA). The effectiveness of the extraction was confirmed by electrophoresis on 1% (w/v) agarose gel at 85 V for 40 min. The quantitation and purity of the extracted DNA were determined in a spectrophotometer for microsamples (NanoDrop 2000, Thermo Scientific, USA). Procedures for DNA amplification and separation by DGGE, sequencing, and 16S rRNA sequencing and data analysis are described in the Supplementary information section.

### Ecological indices

Based on the DGGE profiles, ecological indices such as species richness (*S*), which characterizes the total number of bands present in a lane, and evenness (*J*), which exposes an outline of the predominance of species within the community, were calculated as reported elsewhere (Martínez‐Jardines et al. 2021). Each band in the DGGE gel was considered a fragment of a different species, while bands with similar migration positions were considered fragments of the same species (Silva et al. 2014; Martínez‐Jardines et al. 2021). The relation between the surface area of a band and its mean pixel intensity was considered the intensity of the band (proportion of individuals) and was calculated using the image analysis program ImageJ (https://imagej.net/ij/index.html), developed at the National Institutes of Health.

### Statistical analysis

The relationship among the ecological indices (*S* and *J*), intensity of the band (proportion of individuals), and the physiologic (in terms of consumption efficiencies (ENH_4_^+^-N, E2-CP-C) and yield values (YNO_3_^−^-N)) and kinetic performance (in terms of specific rates (qNH_4_^+^-N, q2-CP-C, qNO_3_^−^-N)) of the nitrifying process was established by means of Pearson correlation analysis. All statistical analyses were performed with the NCSS 2020 software (version 20.0.3).

## Results

### Physiologic and kinetic behavior of nitrifying sludge during cometabolic 2-CP consumption in SBR

The evaluation of the cometabolic consumption of 2-CP by a nitrifying sludge in the SBR fed with 60 mg/L 2-CP-C and different initial NH_4_^+^ concentrations (100, 200, 300, 400, and 500 mg/L NH_4_^+^-N) has been previously reported by Martínez-Jardines et al. (2018). A short summary is presented as follows: for more detailed information, please refer to Martínez-Jardines et al. (2018). Throughout the 13 operational cycles (reaction time between 14 and 21 days) and irrespective of the increase in NH_4_^+^ concentration, the sludge achieved complete nitrification in 14 days, wherein the NH_4_^+^ was completely oxidized to NO_3_^−^ (ENH_4_^+^-N close to 99% and YNO_3_^−^-N between 0.93 and 0.99 g NO_3_^−^-N/g NH_4_^+^-N consumed). In cycle 13 (14 days of reaction time) fed with 500 mg/L NH_4_^+^-N, the nitrifying sludge was able to completely consume 2-CP within the first 7 days of reaction, accounting for an E2-CP-C of 100%. The increase in NH_4_^+^ concentration from cycle 1 to cycle 13 provoked an increment in the qNH_4_^+^-N and q2-CP-C up to 5.2 and 3.1 times, respectively. A direct and significant relationship between qNH_4_^+^-N and q2-CP-C with a correlation coefficient (*r*) of 0.83 was established, evidencing the effect of the increase in NH_4_^+^ concentration on 2-CP cometabolic consumption. Participation of the AMO in the 2-CP consumption was evidenced in batch assays conducted with 2-CP, NH_4_^+^, and ATU as a specific inhibitor of the AMO enzyme, and the sludge inoculated into the reactors. Moreover, an increase in the AMO enzyme participation in the 2-CP consumption was determined after 13 operating cycles in the SBR.

### Population dynamics of nitrifying sludge in the presence of 2-CP and different initial ammonium concentrations in SBR

**Fig. 1:**
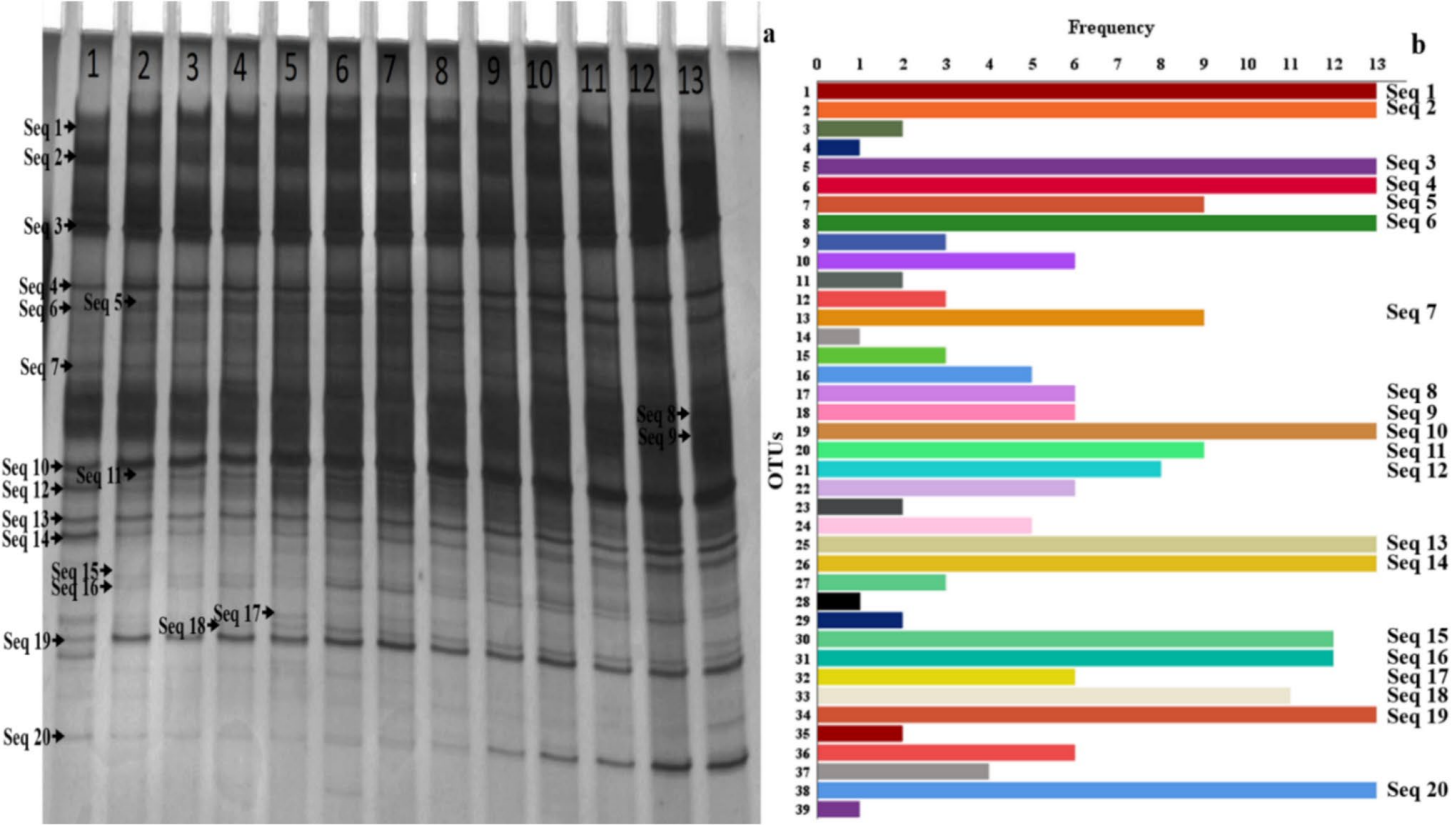
**a** DGGE analysis of the V6–V8 regions of 16S rDNA of the nitrifying sludge throughout the operating cycles with 60 mg of 2-CP-C/L and with different initial ammonium concentrations. Lane 1 with 100 mg of NH_4_^+^-N/L (cycle 1 of operation); lanes 2, 3, and 4 with 200 mg of NH_4_^+^-N/L (cycles 2, 3, and 4 of operation, respectively); lanes 5, 6, and 7 with 300 mg of NH_4_^+^-N/L (cycles 5, 6, and 7 of operation, respectively); lanes 8, 9, and 10 with 400 mg of NH_4_^+^-N/L (cycles 8, 9, and 10 of operation, respectively); lanes 11, 12, and 13 with 500 mg of NH_4_^+^-N/L (cycles 11, 12, and 13 of operation, respectively). **b** Frequency analysis of the appearance of nitrifying sludge species throughout the operating cycles with 60 mg of 2-CP-C/L and with different initial ammonium concentrations. The cut and sequenced bands are indicated in the figure

In the DGGE band profile of the nitrifying sludge throughout the operation cycles, a total of 276 bands were found, of which 39 were identified as different species (Fig. 1). The analysis of the frequency of species appearance in the nitrifying sludge showed that 10 species were found in all the cycles, 7 species appeared with a frequency of 8 to 12 times, and 22 species were found between 1 and 7 times (Fig. 1b). These results indicate that only 25.6% of the species detected in the DGGE were observed since the experimentation beginning with 60 mg/L 2-CP-C and 100 mg/L NH_4_^+^-N and remained present. The remaining 74.4% of the species were detected at one or more sampling points throughout the cycles, indicating a change in the abundance of nitrifying sludge populations, caused by the increase in NH_4_^+^ concentration and the presence of 2-CP.

According to the intensity of the DGGE bands, the proportion of individuals was estimated to assess the population dynamics through a heat map (Liu et al. 2010; Neilson et al. 2013) (Supplementary information, Fig. S1). The pattern of intensities of the bands was analyzed using the ecological indices *S* and *J* (Table 1). In the initial cycle with 100 mg/L NH_4_^+^-N, the nitrifying sludge presented an *S* value of 22. With the increase to 200 mg/L NH_4_^+^-N, *S* increased to 25. However, after two cycles with the same NH_4_^+^ concentration, this index decreased to 23. A similar behavior was obtained with concentrations of 300 and 400 mg/L NH_4_^+^-N. While in the operating cycles with 500 mg/L NH_4_^+^-N, the trend was different as the *S* value increased from 18 to 22 in cycle 13. On the other hand, the *J* index tended to increase. In the first cycle, the *J* index was 0.89, whereas in the last cycle with 500 mg/L NH_4_^+^-N, it reached a value of 0.99.

**Table 1:** Ecological indices obtained from the DGGE gel of the nitrifying sludge throughout the operating cycles with 60 mg/L 2-CP-C and different initial NH_4_^+^ concentrations: Indices of species richness (*S*) and evenness (*J*) of the nitrifying microbial community

Ammonium concentration (mg N/L)													
	100	200			300			400			500		
Cycle	1	2	3	4	5	6	7	8	9	10	11	12	13
S	22	25	20	23	25	24	21	22	19	18	18	17	22
J	0.89	0.88	0.92	0.95	0.93	0.93	0.93	0.94	0.96	0.96	0.95	0.97	0.99

**Fig. 2:**
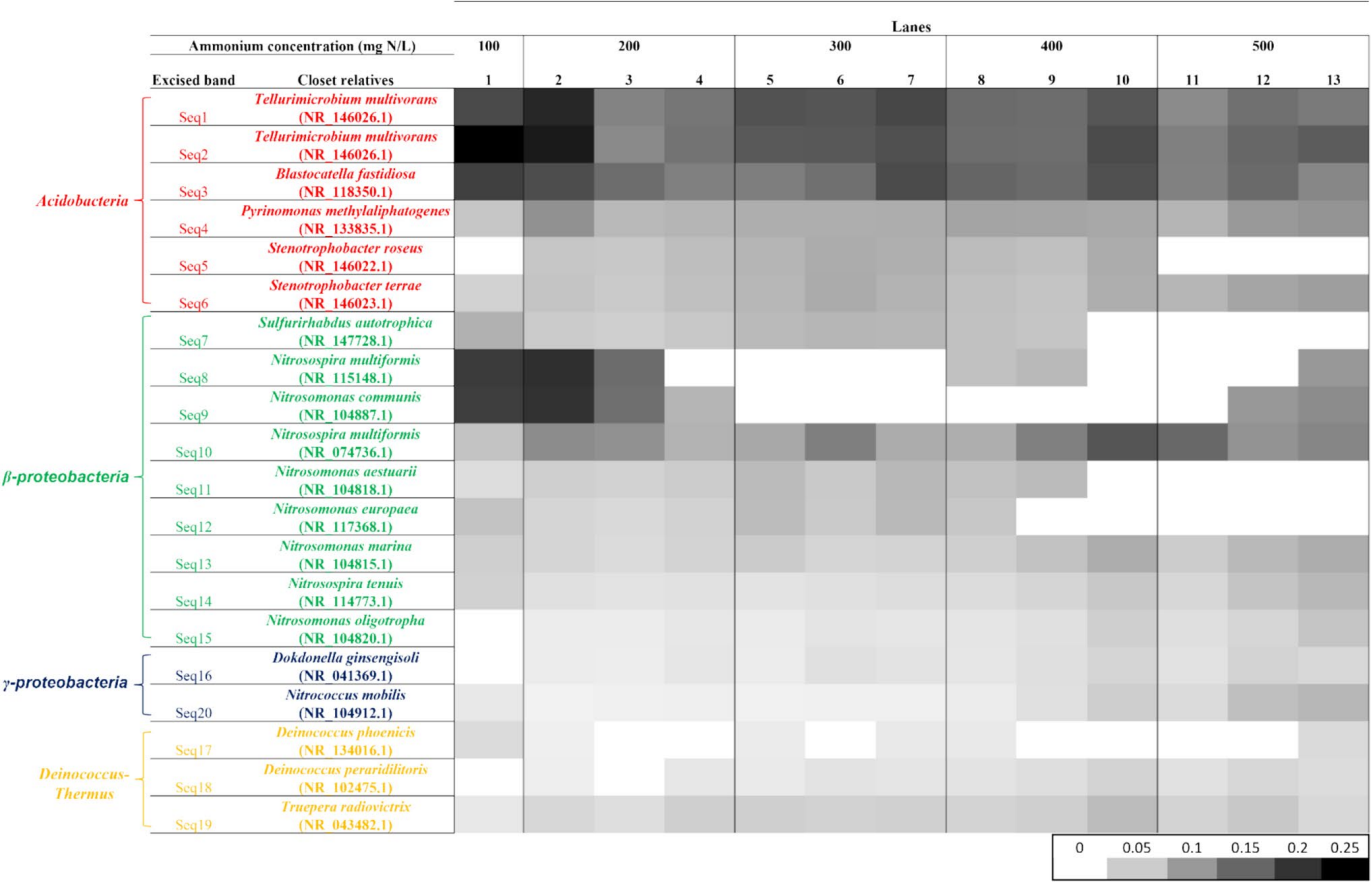
Heat map of the band intensities of the 20 sequences obtained from the DGGE with its related species throughout the operation cycles with 60 mg of 2-CP-C/L and with different initial ammonium concentrations of the SBR

Twenty bands of the DGGE gel were sequenced, corresponding to the bands with the highest frequency (Fig. 1). The taxonomic assignment was made using the NCBI GenBank bacterial 16S rRNA database (Supplementary information, Fig. S2a). The sequences of the organisms with the greatest similarity obtained by the BLAST analysis were used for the phylogenetic analysis. The sequenced bands were grouped as Proteobacteria (45% as β-proteobacteria and 10% as γ-proteobacteria class), Acidobacteria (30%), and Deinococcus-Thermus (15%) phyla (Fig. S2b). Eight of the sequenced species were associated with AOB bacteria (Seq 8–15), most belonging to the *Nitrosomonas* and *Nitrosospira* genera (Fig. 2). The maintenance of these sequences along the operation cycles evidences their tolerance to 2-CP presence. All these sequences were at the beginning of the SBR, except Seq 15, which was not detected in cycle 1 of operation. Seq 15, related to *Nitrosomonas oligotropha*, was detected by increasing the NH_4_^+^ concentration to 200 mg/L NH_4_^+^-N and had an increasing trend in subsequent operating cycles. Seq 10 was associated with *Nitrosospira multiformis*. Its population was unstable, since it increased and decreased throughout the operation cycles. The species of Seq 13 related to *Nitrosomonas marina*, Seq 14 related to *Nitrosospira tenuis*, and Seq 20 related to *Nitrococcus mobilis* appeared in all the operation cycles of the SBR regardless of the increase in the NH_4_^+^ concentration and the constant exposure to 2-CP. Despite a decrease in their individual proportion in cycle 2 of operation (200 mg/L NH_4_^+^-N) which was maintained until cycle 8, a general tendency to increase their population was favored by the increase in NH_4_^+^ (400–500 mg/L NH_4_^+^-N), evidencing their adaptation to high NH_4_^+^ concentration environments. It is remarkable that Seq 20 related to *Nitrococcus mobilis* is a NOB belonging to a γ-proteobacteria phylum.

Six members of the Acidobacteria phylum were identified, associated with different heterotrophic species. Five of these species (Seq 1, 2, 3, 4, and 6) appeared since the first operation cycle, and similar to some of the AOB species, they were maintained until the end of the experimentation, evidencing their tolerance to 2-CP and high NH_4_^+^ concentrations. The species of Seq 1 and 2 were related to *Tellurimicrobium multivorans*, whereas Seq 3, Seq 4, and Seq 6 were, respectively, related to *Blastocatella fastidiosa*, *Pyrinomonas methylaliphatogenes*, and *Stenotrophobacter terrae*. Sequences 1, 2, and 3 were the dominant heterotrophic species due to their high individual proportion in cycle 1 compared to the other species found in the DGGE. Subsequently, their proportion decreased with the increase in the NH_4_^+^ concentration, keeping oscillating throughout the cycles and having a tendency to decrease in the last cycles with the highest NH_4_^+^ concentration of 500 mg/L NH_4_^+^-N. Sequences of Seq 17, 18, and 19 were grouped in the Deinococcus-Thermus phylum. They had a high similarity with the *Deinococcus phoenicis*, *Deinococcus peraridilitoris*, and *Truepera radiovictrix* species, respectively. In contrast to the tolerance to all NH_4_^+^ concentrations of Seq 19, Seq 17 and Seq 18 species were not detected in all operation cycles, but they were present in the last operation cycle with 500 mg/L NH_4_^+^-N, demonstrating their adaptation to the presence of high NH_4_^+^ concentrations. Finally, two members of the γ-proteobacteria class were also detected, Seq 16 related to *Dokdonella ginsengisoli* and Seq 20 related to *Nitrococcus mobilis*.

### Bacterial community structure of nitrifying sludge in the presence of 2-CP and different initial ammonium concentrations in the SBR

**Fig. 3:**
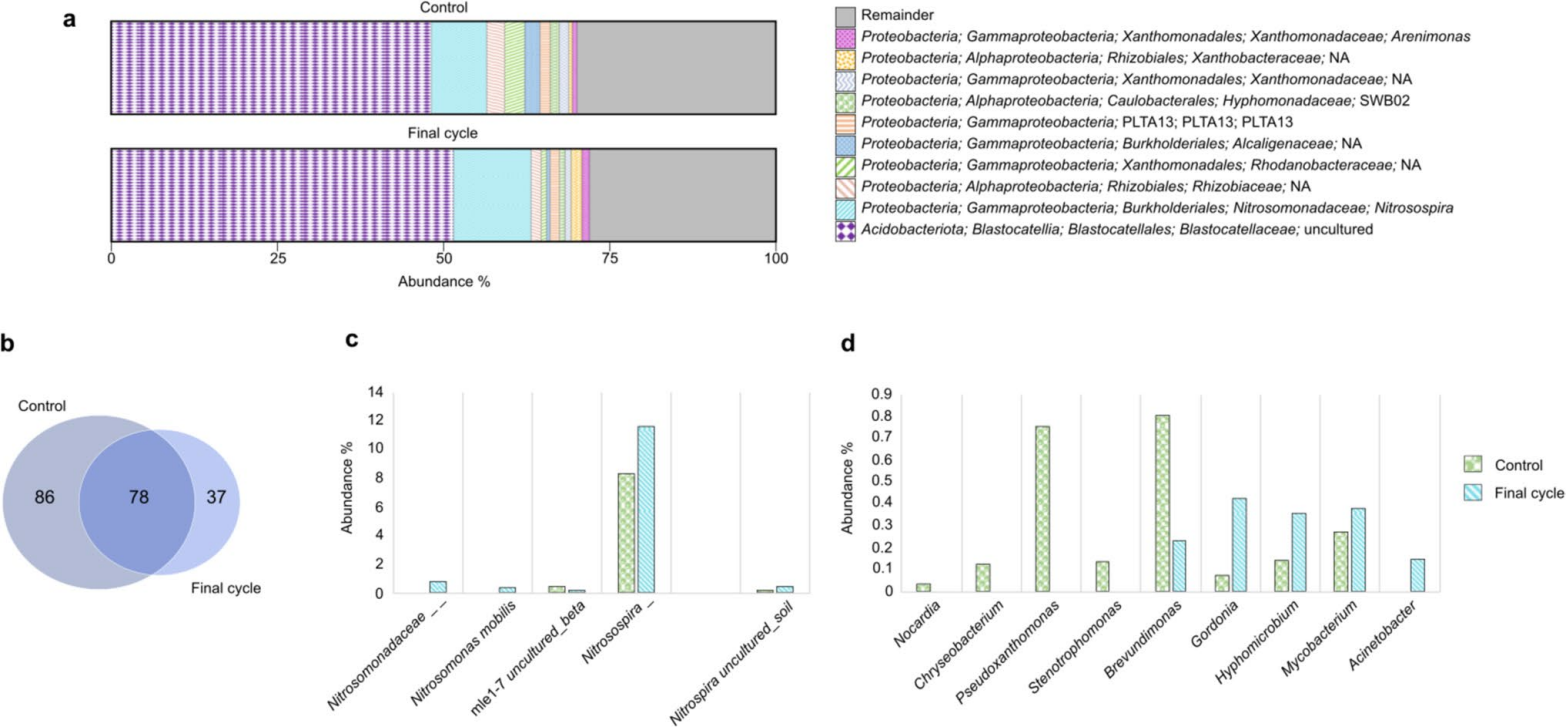
Relative abundance of nitrifying sludge at the beginning and end of the experiment. **a** Abundance relative to phylum level. **b** The Venn diagram represents the bacteria detected at the beginning and end of the experiment; the intersection of the circles indicates the species of bacteria detected at both sampling points. **c** Relative abundance of AOB and NOB species. **d** Relative abundance of heterotrophic bacteria previously identified as biodegraders of phenolic compounds

Samples from cycle 1 (control) and cycle 13 (final cycle) were used to evaluate the changes in the abundance of the microorganisms present at the beginning and end of the experimentation through the sequencing of the V4 region of the 16S rRNA gene (Fig. 3a). A total of 201 different OTUs were identified, which 86 species were only detected at the beginning of the experiment with an NH_4_^+^ concentration of 100 mg/L NH_4_^+^-N and 37 species were only detected at the end of the experiment with exposure to 500 mg/L NH_4_^+^-N (Fig. 3b). On the other hand, 78 different species were detected in both samples. In this group, AOB bacteria of the *Nitrosospira* genus were found, occupying the second place of the most abundant species in the nitrifying sludge, and the *mle 1–7* genus belonging to the Nitrosomonadaceae family with an abundance of less than 0.5% (Fig. 3c).

*Nitrosomonas mobilis* was detected at the end of the experiment with an abundance of ~ 0.37% and another species belonging to the family Nitrosomonadaceae with an abundance of ~ 0.8%. In contrast, only one uncultivated species of the *Nitrospira* genus (NOB) was identified in both samples. Some genera of heterotrophic bacteria previously associated with the consumption of phenolic compounds were identified. The genera *Nocardia*, *Chryseobacterium*, *Pseudoxanthomonas*, and *Stenotrophomonas* were detected only at the beginning of the experiment with an abundance of less than ~ 1% (Fig. 3d). While at the end of the experiment, only the *Acinetobacter* genus was detected with ~ 0.1% abundance. On the other hand, the genera *Brevundimonas*, *Gordonia*, *Hyphomicrobium*, and *Mycobacterium* kept present from the beginning to the end of the experiment. These species increased in abundance at the end of the experimentation with the exception of *Brevundimonas*, which was the only genus that had a decrease in abundance.

### Relationship of the physiologic and kinetic response of nitrifying sludge with its population dynamics

As the operation cycles went by, kinetic (in terms of specific rates, *q*) and physiologic (in terms of NH_4_^+^ and 2-CP consumption efficiencies, *E*, and product formation yield, *Y*) behavior of the nitrifying sludge was associated with the sludge homogeneity and richness (in terms of ecological indices, *J* and *S*, respectively) and the bacterial proportion of the species present in the nitrifying consortium through a statistical analysis of correlations. This was done by associating the increase in band intensity, interpreted as an increase in the proportion of individuals, with the nitrifying response. It is important to emphasize that only the species that were present in most of the operation cycles were taken into account to evaluate the relationship between the nitrifying response and the proportion of species since they were the most tolerant species to the presence of NH_4_^+^ and 2-CP. Thus, they could be participating in the consumption of both compounds throughout the study. However, it is not ruled out that the rest of the species might be participating in the respiratory process performance and/or in 2-CP consumption. Likewise, only the cases in which the correlations were significant and positive are presented. Pearson’s correlation coefficient (*r*) and statistical significance values obtained are shown in a correlation matrix (Table 2).

**Table 2:** Correlation matrix (upper Pearson’s correlation coefficient (*r*) and lower significance) of the operating cycles, the initial concentration of NH_4_^+^, the kinetic and physiological response variables of the nitrifying respiratory process, the consumption of 2-CP with the ecological indices, and proportion of individuals of different species obtained from DGGE sequencing

	Index		Acidobacteria	β-Proteobacteria				Deinococcus-Thermus		γ-Proteobacteria	
	*S*	*J*	*Stenotrophobacter terrae* (NR_146023.1)	*Nitrosospira multiformis* (NR_074736.1)	*Nitrosomonas marina* (NR_104815.1)	*Nitrosospira tenuis* (NR_114773.1)	*Nitrosomonas oligotropha* (NR_104820.1)	*Truepera radiovictrix* (NR_043482.1)	*Deinococcus peraridilitoris* (NR_102475.1)	*Dokdonella ginsengisoli* (NR_041369.1)	*Nitrococcus mobilis* (NR_104912.1)
Cycles	− 0.614^*^	0.891^**^	0.783^**^	0.551	0.763^**^	0.705^**^	0.887^**^	0.556^*^	0.847^**^	0.862^**^	0.815^**^
	0.030	0.001	0.002	0.051	0.002	0.007	0.001	0.048	0.001	0.001	0.001
[NH_4_^+^]	− 0.565^*^	0.823^**^	0.739^**^	0.566^*^	0.680^*^	0.600^*^	0.834^**^	0.542	0.824^**^	0.825^**^	0.738^**^
	0.040	0.001	0.004	0.044	0.011	0.030	0.001	0.056	0.001	0.001	0.004
ENH_4_^+^-N	− 0.410	0.641^*^	0.665^*^	0.569^*^	0.425	0.227	0.732^**^	0.668^*^	0.730^**^	0.819^**^	0.408
	0.170	0.020	0.013	0.042	0.148	0.455	0.004	0.013	0.005	0.001	0.167
YNO_3_^−^-N	− 0.150	0.620^*^	0.650^*^	− 0.065	0.505	0.347	0.494	0.550	0.587^*^	0.561^*^	0.390
	0.630	0.020	0.016	0.833	0.079	0.246	0.086	0.052	0.035	0.046	0.187
qNH_4_^+^-N	− 0.510	0.816^**^	0.800^**^	0.548	0.693^**^	0.634^*^	0.826^**^	0.449	0.725^**^	0.756^**^	0.767^**^
	0.080	0.001	0.001	0.053	0.009	0.020	0.001	0.124	0.005	0.003	0.002
qN-NO_3_^−^-N	− 0.360	0.601^*^	0.460	0.295	0.128	0.044	0.574^*^	0.427	0.381	0.656^*^	0.206
	0.230	0.030	0.113	0.328	0.678	0.887	0.040	0.146	0.200	0.015	0.500
E2-CP-C	− 0.450	0.280	0.328	0.334	0.350	0.370	0.209	0.038	0.080	0.188	0.378
	0.130	0.350	0.273	0.264	0.241	0.213	0.493	0.902	0.794	0.539	0.203
q2-CP-C	− 0.654^*^	0.855^**^	0.592^*^	0.550	0.647^*^	0.518	0.780^**^	0.600^*^	0.815^**^	0.793^**^	0.624^*^
	0.020	0.001	0.033	0.052	0.017	0.070	0.002	0.030	0.001	0.001	0.023

Significant correlations are indicated in bold. An asterisk (*) next to the value of *r* indicates that the correlation is significant with an α value of 0.05 and two asterisks (**) with an α value of 0.01. Thirteen data points were used for each correlation analysis and represent the number of operating cycles performed on the SBR. *r* can take values between − 1 and 1

**Fig. 4:**
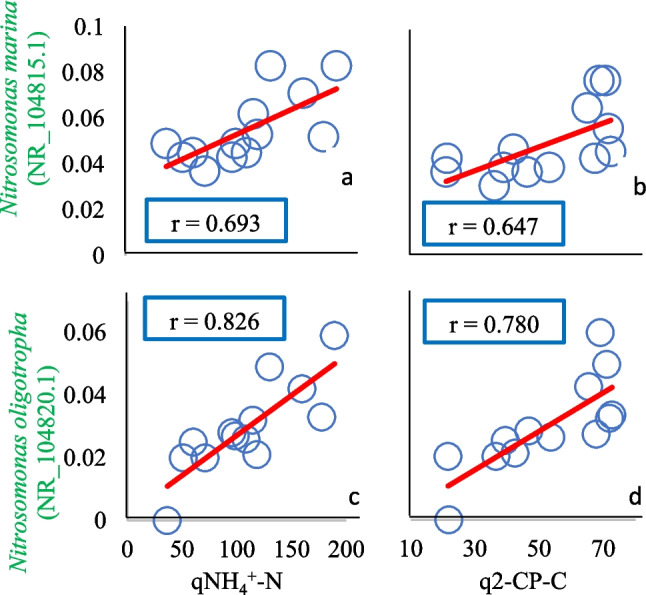
Correlation profiles of the band intensities of *Nitrosomonas marina* with the consumption specific rate of ammonium (**a**) and the consumption specific rate of 2-CP (**b**). *Nitrosomonas oligotropha* with the consumption specific rate of ammonium (**c**) and the consumption specific rate of 2-CP (**d**). *r* = Pearson correlation coefficient

It was found that the increase in the NH_4_^+^ concentration and the course of the operating cycles was associated with the decrease in the number of species of the nitrifying sludge so that the sludge species richness (*S*) decreased throughout the experimentation. The *J* index was associated with most of the physiologic and kinetic nitrifying response variables showing a direct, proportional, and significant correlation with *r* coefficients between 0.60 and 0.81. Thus, as the operation cycles elapsed and the NH_4_^+^ concentration increased, the evenness of the nitrifying sludge increased, so the nitrifying sludge tended to be homogeneous. Under these conditions, very efficient and complete nitrification, as well as increases of 5.1 and 5.2 times in qNH_4_^+^-N and qNO_3_^−^-N, was respectively obtained. Most of the band intensities of the species obtained had a significant and directly proportional correlation with the operating cycles and/or with the increase in NH_4_^+^ concentration. In fact, four AOB bacteria species (*Nitrosospira multiformis*, *Nitrosomonas marina*, *Nitrosospira tenuis*, and *Nitrosomonas oligotropha*) had a significant and directly proportional correlation with the NH_4_^+^ concentration (*r* between 0.56 and 0.83) and three of them (*Nitrosomonas marina*, *Nitrosospira tenuis*, and *Nitrosomonas oligotropha*) with the number of operating cycles (*r* between 0.70 and 0.88). A positive and significant relationship was found between the AOB species *Nitrosospira multiformis* and *Nitrosomonas oligotropha* with the high ENH_4_^+^-N; *Nitrosomonas marina*, *Nitrosospira tenuis*, and *Nitrosomonas oligotropha* with the increment in qNH_4_^+^-N; as well as *Nitrosomonas marina* and *Nitrosomonas oligotropha* with the increment in q2-CP-C. The increase in the proportion of individuals of these two AOB species, *Nitrosomonas marina* and *Nitrosomonas oligotropha*, presented a directly proportional relationship with the increment in both qNH_4_^+^-N and q2-CP-C values (Fig. 4). Likewise, regarding the AOB bacteria identified in the SBR, it was found that the band intensity of *Nitrosomonas oligotropha* had a significant and directly proportional relationship with ENH_4_^+^-N, qNH_4_^+^-N, and q2-CP-C values. Moreover, a significant and directly proportional relationship between the heterotrophic species and the increase in q2-CP-C can be observed in the correlation matrix for the species *Dokdonella ginsengisoli* of the γ-proteobacteria class, *Deinococcus peraridilitoris* and *Truepera radiovictrix* of the Deinococcus-Thermus phylum, and *Stenotrophobacter terrae* from the Acidobacteria phylum, indicating the association of the individuals increase of these heterotrophic species with the increase in q2-CP-C.

## Discussion

Results obtained by the DGGE, sequencing analysis, and the ecological indices indicated the presence of different species with changing abundance during the cometabolic 2-CP consumption with increasing NH_4_^+^ concentrations throughout the operation cycles in the nitrifying SBR. The *S* values obtained in the present work at the end of the experimentation indicated a decrease in the richness of the nitrifying community, whereas the *J* index close to 1 evidenced that the microbial community was uniform, namely, there was no predominance of species within the microbial community of the nitrifying sludge. It should be remembered that in the present study, ENH_4_^+^-N close to 100%, YNO_3_^−^-N around 1, and E2-CP-C of 100% were obtained. The high consumption efficiency of both nitrogenous and carbonaceous compounds, as well as a homogeneous microbial community, coincides with that reported by several authors, who suggest that the use of SBR systems could lead to obtaining a steady nitrifying process throughout the operating cycles even in the presence of recalcitrant compounds such as *p*-cresol (Silva et al. 2014) or 2-CP (Martínez‐Jardines et al. 2021). According to the correlation analysis, the results evidenced that the metabolic (ENH_4_^+^-N, YNO_3_^−^-N) and kinetic (qNH_4_^+^-N, qYNO_3_^−^-N) behavior was related to the evenness or homogeneity (*J*) of the nitrifying sludge, but these variables were not related to the number of species (*S*) present in the nitrifying community. Likewise, 2-CP consumption efficiency (E2-CP-C) was neither related to the microbial richness (*S*) nor to its evenness. The results of the present work contrast with those reported in nitrifying SBR fed with *p*-cresol (Silva et al. 2014) wherein the authors reported a *J* index of 0.68 ± 0.03 with a stable consortium with the predominance of some species which could be related to the high stability of the nitrifying process (ENH_4_^+^-N and YNO_3_^−^-N of 99.0% ± 0.5% and 0.98 ± 0.07, respectively). However, there is a coincidence with those reported by Martínez‐Jardines et al. (2021), as the authors indicated that regardless of the elapsed operation cycles and the increase in 2-CP concentration, a *J* index of 0.98 ± 0.07 pointed out no species predominance in the microbial community of the nitrifying sludge. In contrast, these authors reported the development of a more diverse bacterial community in SBR resulting in full 2-CP consumption and minimal changes in q2-CP-C, as well as a substantial adverse effect of 2-CP on nitrifying metabolism (incomplete nitrification in some operating cycles). Remarkably, in the present work, a direct, proportional, and significant correlation of the 2-CP consumption rate with the *J* index evidenced the relationship between the increase in q2-CP-C tendency with the homogeneity of the nitrifying consortium (*r* = 0.85). In contrast, an inversely proportional and significant correlation (*r* =  − 0.65) between q2-CP-C and the *S* index evidenced the relationship between the increase in q2-CP-C and the diminishment in species richness. These results suggest that the increase in q2-CP-C throughout the operating cycles could be related to the decreased tendency of the species richness (*S*) and the increased tendency in homogeneity (*J*) of the nitrifying consortium. In this sense, it is possible that as operation cycles elapsed in the present study, the non-predominant remaining microorganisms were able to fulfill the nitrification respiratory process completely and efficiently with an improvement in the kinetic activity in the NH_4_^+^ and 2-CP consumption and NO_3_^−^ production rate.

Regarding the microbial community composition, the AOB found in the nitrifying sludge belong to the Nitrosomonadaceae family whose genera have been reported in different operational conditions (Zhang et al. 2019b), environmental conditions (Soliman and Eldyasti 2018), and different feeding NH_4_^+^ concentrations (Martínez‐Jardines et al. 2021). Among the genera of the Nitrosomonadaceae family, the *Nitrosomonas* and *Nitrosospira* genera stood out. The presence of *Nitrosomonas marina* has been reported in nitrifying sludge from two wastewater treatment plants (WWTPs), a municipal WWTP that treats 20 mg NH_4_^+^-N/L and a swine WWTP that treats 220 mg NH_4_^+^-N/L (Whang et al. 2009), while *Nitrosospira tenuis* and *Nitrosospira multiformis* species have been found in a laboratory-scale aerobic granular sludge reactor (Winkler et al. 2013). It is noticeable that *Nitrosomonas oligotropha* was maintained in 12 of the 13 cycles of operation, so this species resulted to be tolerant to high NH_4_^+^ concentrations and the presence of 2-CP. Regarding NOB bacteria, it is interesting to note that the presence of *Nitrococcus mobilis* of the *Nitrococcus* genus and the Ectothiorhodospiraceae family (Watson and Waterbury 1971) has only been reported in marine systems (Füssel et al. 2017) and not in wastewater treatment or nitrifying processes, so to the best of the authors’ knowledge, this is the first time that this bacterium has been observed in a nitrifying reactor fed with 2-CP and different NH_4_^+^ concentrations. Meanwhile, the species of the genus *Nitrospira* have been reported in other studies with nitrifying sludge (Martínez‐Jardines et al. 2021). It is remarkable that despite using two different methodologies in the present work, only two NOB species were detected. Thus, regardless that their abundance was below 0.5%, these both species may be responsible for carrying out the nitrite-oxidizing process and obtaining a complete nitrification throughout the experiment.

Concerning the heterotrophic community, the growth of some heterotrophic genera such as *Gordonia*, *Hyphomicrobium*, and *Mycobacterium* was favored by the NH_4_^+^ concentration increase in the presence of 2-CP, as their abundance augmented at the end of the experimentation. However, the decrease in the proportion of individuals of all heterotrophic species in cycle 13 with respect to cycle 1 indicates that the increase in NH_4_^+^ concentration throughout the operation cycles as well as the constant exposure to 2-CP affected their population. The consumption of hydrocarbons and aromatic compounds such as phenols and cresols, by aerobic heterotrophic bacteria, has been reported in the literature (Das and Kazy 2014). Likewise, improvements in 4-CP consumption alongside increases in heterotrophic strains in soil samples have been registered (Nowak and Mrozik 2018). The authors suggest that the induction of enzymes involved in the degradation of aromatic compounds could be carried out. Members of the *Gordonia* and *Mycobacterium* genera have been isolated from aquatic environments and evaluated as degraders of toxic compounds such as phenol and hydrocarbons (Kim et al. 2009; Arora and Bae 2014; Mai et al. 2021), while members of the genus *Hyphomicrobium* have been found as dominant genera in media containing phenolic compounds such as nonylphenol and octylphenol (Montenegro et al. 2021). Thus, 2-CP consumption might be attributed to the tolerance of these species to the phenolic compound besides their ability to use it as an energy and carbon source. The presence of the *Stenotrophobacter* genus has been reported in activated sludge in an SBR fed with synthetic wastewater with 30.6 ± 0.5 mg NH_4_^+^-N/L (Li et al. 2019). Although the Acidobacteria phylum has been reported in different municipal wastewater treatment plants (Zhang et al. 2019a), textiles (Meerbergen et al. 2017), and petrochemicals (Yang et al. 2015), which can contain NH_4_^+^ and/or phenolic compounds, none of these species has been reported under conditions similar to those of this study, so this is the first time that their tolerance to high NH_4_^+^ concentrations and up to 60 mg 2-CP-C/L has been reported. This suggests their participation in the consumption of both compounds. Despite aerobic heterotrophic bacteria having been reported to be less sensitive to 2-CP toxicity compared to nitrifying bacteria such as *Nitrosomonas* (Blum and Speece 1991), until now, it has not been reported in the literature the participation and/or presence of *Dokdonella ginsengisoli* and species of the Deinococcus-Thermus phylum in wastewater treatment or in the degradation of any recalcitrant compound, which in the present work appeared to be tolerant of the constant 2-CP presence. Therefore, the relationship between the increase in the proportion of the entire heterotrophic species with the increase in q2-CP-C could be attributed both to the tolerance of these species to NH_4_^+^ and 2-CP and to the increase in their population and, consequently, to the increased formation of enzymes capable of 2-CP biotransformation.

The role of ammonium-oxidizing bacteria in 2-CP consumption has also to be considered. According to the results obtained in this study, the increase of NH_4_^+^ concentration up to 500 mg/L NH_4_^+^-N could have favored the increase in band intensity of some individuals of AOB species in the SBR. In the present work, the increase in the proportion of individuals of the AOB species (*Nitrosospira multiformis*, *Nitrosomonas marina*, *Nitrosospira tenuis*, and *Nitrosomonas oligotropha*) could contribute both to the NH_4_^+^ and 2-CP cometabolic consumption and in the improvement of qNH_4_^+^-N and q2-CP-C. In fact, the high and significant correlation (*r* = 0.83) between qNH_4_^+^-N and q2-CP-C could be explained by the increase in the proportion of individuals of *Nitrosomonas oligotropha* and *Nitrosomonas marina*, which could result in higher AMO enzyme levels favoring ammonium oxidation and cometabolic consumption of 2-CP. On the other hand, *Nitrosomonas marina* and *Nitrosomonas oligotropha* showed the strongest relationship with qNH_4_^+^-N and q2-CP-C (Fig. 4), suggesting that according to Martínez-Jardines et al. (2018), the increase in these two species could be associated with the cometabolic consumption of 2-CP in the nitrifying SBR. It has also been reported that by increasing the NH_4_^+^ concentration from 100 to 500 mg/L NH_4_^+^-N in *Nitrosomonas* cultures, the qNH_4_^+^-N and trichloroethylene consumption rate increased (Alpaslan Kocamemi and Çeçen 2007; Kocamemi and Çeçen 2010), wherein the oxygenase production contributed to the trichloroethylene cometabolic consumption. In this sense, it has been suggested that microbial oxygenases catalyze the oxidation of chlorinated solvents and favor their conversion to easily mineralizable products (Frascari et al. 2015). Correlation values of the band intensities of nitrifying and heterotrophic species with the NH_4_^+^ concentration and the elapsed operation cycles registered in the present work suggest that a selection of both heterotrophic and nitrifying microbial species which increased throughout the operation cycles within the SBR occurred.

In brief, this work provides, for the first time, information that demonstrates through statistical correlations the relationship among physiology, kinetics, and population dynamics of a nitrifying sludge, particularly of nitrifying bacteria species such as *Nitrosomonas marina* and *Nitrosomonas oligotropha*, during the cometabolic consumption of recalcitrant compounds such as 2-CP.

Finally, this study examined the population dynamics of a nitrifying sludge with increasing NH_4_^+^ concentrations and 2-CP presence in an SBR. The most prevalent bacterial groups were Proteobacteria (55%) and Acidobacteria (30%) phyla. The 2-CP cometabolic consumption capacity of the nitrifying community was mostly associated to the AOB species of *Nitrosomonas marina* and *Nitrosomonas oligotropha* as well as the participation of heterotrophic species of *Dokdonella ginsengisoli*, *Deinococcus peraridilitoris*, *Truepera radiovictrix*, and *Stenotrophobacter terrae*. The increase in the proportion of AOB individuals showed a significant correlation with an increase in qNH_4_^+^-N (*r* ≥ 0.63) and q2-CP-C (*r* ≥ 0.64), while the increase in q2-CP-C correlated with the heterotrophic species q2-CP-C (*r* ≥ 0.59). Under these conditions, a more homogeneous bacterial community with lower richness was developed as operation cycles elapsed, evidencing the establishment of AOB and heterotrophic species tolerant to high NH_4_^+^ and 2-CP presence, capable of conducting an efficient and complete nitrifying process with the total cometabolic consumption of the chlorinated compound. These results may help to understand the behavior and functioning of the microbial consortia involved in biological processes in order to design more stable and efficient treatment systems for the elimination of inhibitory or toxic compounds such as 2-CP.

## Supplementary Information

Below is the link to the electronic supplementary material.

Supplementary file1 (PDF 654 KB)

## Data Availability

The authors declare that the data supporting the findings of this study are available within the article.
