# Supplementary material for: Relationship assessment of microbial community and cometabolic consumption of 2-chlorophenol
Source: Appl Microbiol Biotechnol. 2025 Jan 24;109:22. doi: 10.1007/s00253-025-13403-7 (PMC11761499; doi:10.1007/s00253-025-13403-7)
Supplement: Supplementary file 1 [file 253_2025_13403_MOESM1_ESM.pdf]

Journal: Applied Microbiology and Biotechnology

Relationship assessment of microbial community and cometabolic consumption of 2-chlorophenol

Manucript No. AMBB-D-23-00449R1

Miguel Martínez-Jardines[1,2]; Omar Oltehua-López[1]; Sergio Martínez-Hernández[2]; Anne-Claire Texier[1]; Flor de María Cuervo-López[1*]

[1]Department of Biotechnology, Universidad Autónoma Metropolitana-Iztapalapa. Av. Ferrocarril San Rafael Atlixco 186, Col. Leyes de Reforma 1A Sección. Iztapalapa, CDMX, 09310, México

[2]Institute of Biotechnology and Applied Ecology, Universidad Veracruzana, Av. de las Culturas Veracruzanas 101, Xalapa, 91090, Veracruz, Mexico

*Corresponding author E-mail address: fmcl@xanum.uam.mx 52(55)58046408, Fax number: 52(55)58046407.

Supplementary material

Material and methods

1. *DNA amplification and separation by DGGE*

The V6–V8 regions of the 16S rDNA gene were amplified using the bacterial primers 968F 5'-GAACGCGAAGAACCTTACC-3' with clamp and 1401R 5'-CGGTGTGTACAAGACCC-3' (Silva et al. 2014). The polymerase chain reaction (PCR) amplification was accomplished in a T100™ Thermal Cycler (Bio-Rad Laboratories, Hercules, USA) according to the experimental conditions reported by Martínez-Jardines et al. (2021). The reaction mixture for PCR and purification were performed as described elsewhere (Bejarano Ortiz et al. 2020; Martínez-Jardines et al. 2021). Amplification products were separated by DGGE in a universal DCode mutation detection system (Bio-Rad Laboratories) with TAE 0.5X at 60 °C. Polyacrylamide gels were used at 6% (w/v) and the denaturant gradients varied from 30% to 60% (100% denaturant stock solution was made of 7 mol/L urea and 40% formamide). The gel was run for 5 min at 200 V and then for 16 h at 85 V, according to the procedure described by Silva et al. (2014). DGGE gels were stained with silver nitrate (Cj et al. 1994) and scanned in a MiniLumi (DNR Bio Imaging system).

2. *Sequencing*

Bands from DGGE were excised and reamplified using the same primers (968F without clamp and 1401R). The products of PCR were purified using the Wizard®SV gel and PCR clean-up system Kit

(Promega, Madison, WI, USA) and sequenced (Macrogen Inc. Seoul, South Korea) with the ABI 3730xl DNA Sequencer (Applied Biosystems) using Big-Dye Terminator V1.1 and 3.1 ready reaction cycle sequencing. Sequence data of the amplicons from excised DGGE bands were quality checked and screened for potential chimeras using Bellerophon (Huber et al. 2004) which were excluded from further analysis. The DNA sequences were aligned and analyzed as described elsewhere (Bejarano Ortiz et al. 2020) using the software Basic Local Alignment Search Tool (BLAST) (http://blast.ncbi.nlm.nih.gov/Blast.cgi), the phylogenetic affiliations of the partial sequences were assessed. Phylogenic trees of the 16S rDNA gene sequences of DGGE fragments were constructed using a neighbor-joining method with the Jukes–Cantor distance model and related sequences from NCBI GenBank 16s rDNA database of bacteria as implemented in MEGAX software (Kumar et al. 2018). The robustness of the phylogeny was tested by bootstrap analysis with 1000 iterations (Martínez-Jardines et al. 2021). The identified sequences were deposited in GenBank under accession no. OQ711740-OQ711756.

3. 16S rRNA sequencing and data analysis

The previously purified cycle 1 and 13 DNA samples were used to sequence the V4 region of the 16S rRNA gene using primers 515F and 806R. Paired-end sequencing was performed by the company BGI (Beijing Genome Institute, Shenzhen, China) using the Ilumina paired-end HiSeq 2500 sequencing platform. All bioinformatic analysis were performed using QIIME 2 (Bolyen et al. 2019), default parameters were used for demultiplexing; the DADA2 plugin was used to denoising, the sequences were truncated to a length of 250bp for all reads. A classifier was created using naive Bayes and the ARB-SILVA (version 138) taxonomic classification to carry out the taxonomic assignment. The raw sequences were deposited in GenBank under the Bioproject number PRJNA1044135.

Fig. S1

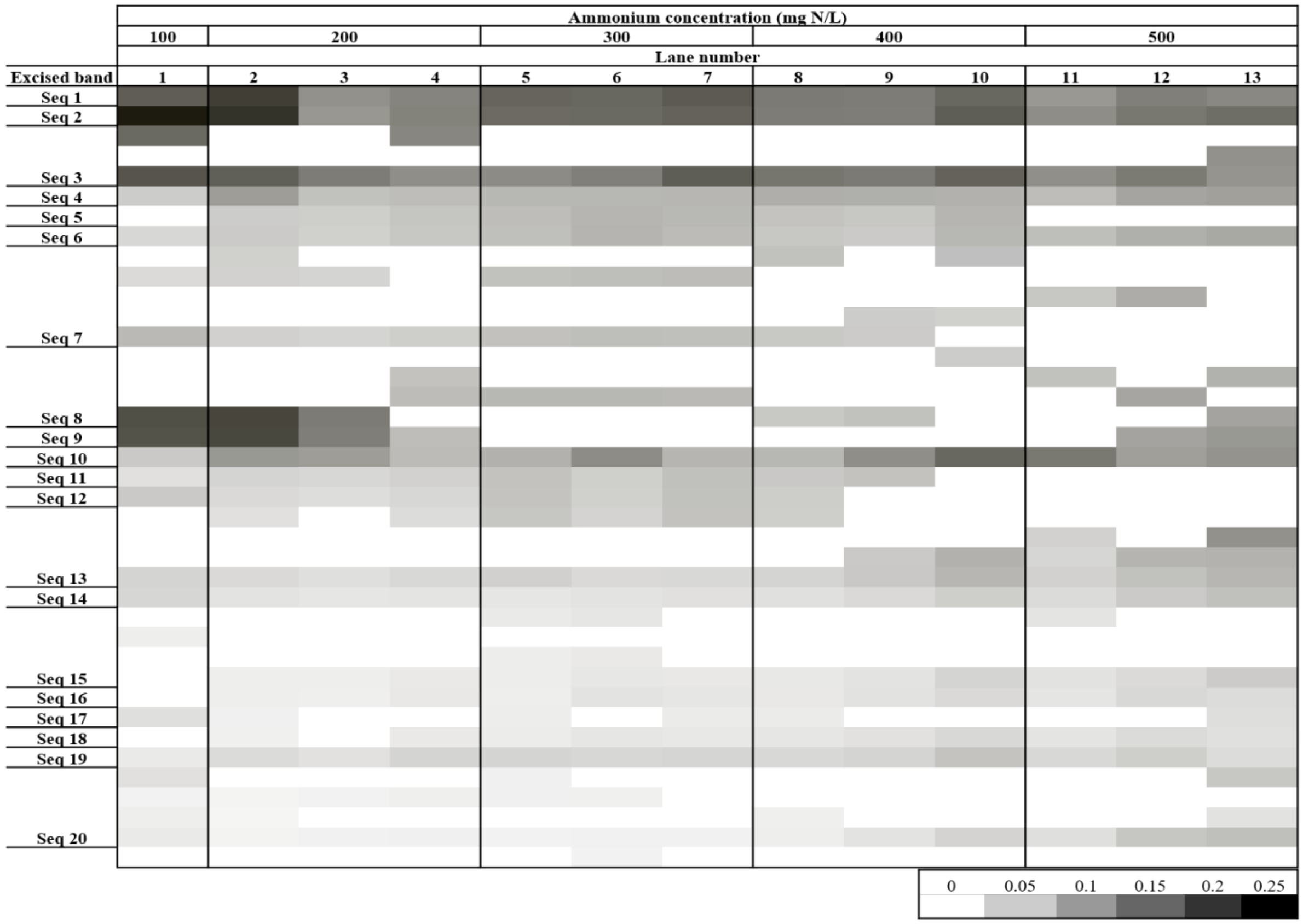
Ammonium concentration (mg N/L)
100
200
300
400
500
Lane number
Excised band
1
2
3
4
5
6
7
8
9
10
11
12
13
Seq 1
Seq 2
Seq 3
Seq 4
Seq 5
Seq 6
Seq 7
Seq 8
Seq 9
Seq 10
Seq 11
Seq 12
Seq 13
Seq 14
Seq 15
Seq 16
Seq 17
Seq 18
Seq 19
Seq 20
0
0.05
0.1
0.15
0.2
0.25

Fig. S2

a

Excised band from DGGE	Closest relatives	% Identity	% Query Cover	E Value
Seq1	*Tellurimicrobium multivorans* (NR_146026.1)	92.79	98	$7e^{-123}$
Seq2	*Tellurimicrobium multivorans* (NR_146026.1)	91.93	100	$5e^{-125}$
Seq3	*Blastocatella fastidiosa* (NR_118350.1)	90.73	100	$2e^{-114}$
Seq4	*Pyrinomonas methylaliphatogenes* (NR_133835.1)	94.9	100	$1e^{-155}$
Seq5	*Stenotrophobacter roseus* (NR_146022.1)	95.87	100	$5e^{-109}$
Seq6	*Stenotrophobacter terrae* (NR_146023.1)	98.71	99	$9e^{-157}$
Seq7	*Sulfurirhabdus autotrophica* (NR_147728.1)	83.84	99	$2e^{-88}$
Seq8	*Nitrosospira multiformis* (NR_115148.1)	99.38	100	$2e^{-168}$
Seq9	*Nitrosomonas communis* (NR_104887.1)	98.88	100	0.0
Seq10	*Nitrosospira multiformis* (NR_074736.1)	98.8	100	$6e^{-169}$
Seq11	*Nitrosomonas aestuarii* (NR_104818.1)	95.52	95	$1e^{-151}$
Seq12	*Nitrosomonas europaea* (NR_117368.1)	100	100	$3e^{-131}$
Seq13	*Nitrosomonas marina* (NR_104815.1)	96.13	100	$2e^{-143}$
Seq14	*Nitrosospira tenuis* (NR_114773.1)	97.64	100	$2e^{-122}$
Seq15	*Nitrosomonas oligotropha* (NR_104820.1)	88.54	80	$1e^{-106}$
Seq16	*Dokdonella ginsengisoli* (NR_041369.1)	97.2	100	$3e^{-172}$
Seq20	*Nitrococcus mobilis* (NR_104912.1)	96.88	100	0.0
Seq18	*Deinococcus peraridilitoris* (NR_102475.1)	92.02	97	$2e^{-128}$
Seq19	*Truepera radiovictrix* (NR_043482.1)	97.7	99	$4e^{-171}$
Seq17	*Deinococcus phoenicis* (NR_134016.1)	82.66	98	$7e^{-89}$

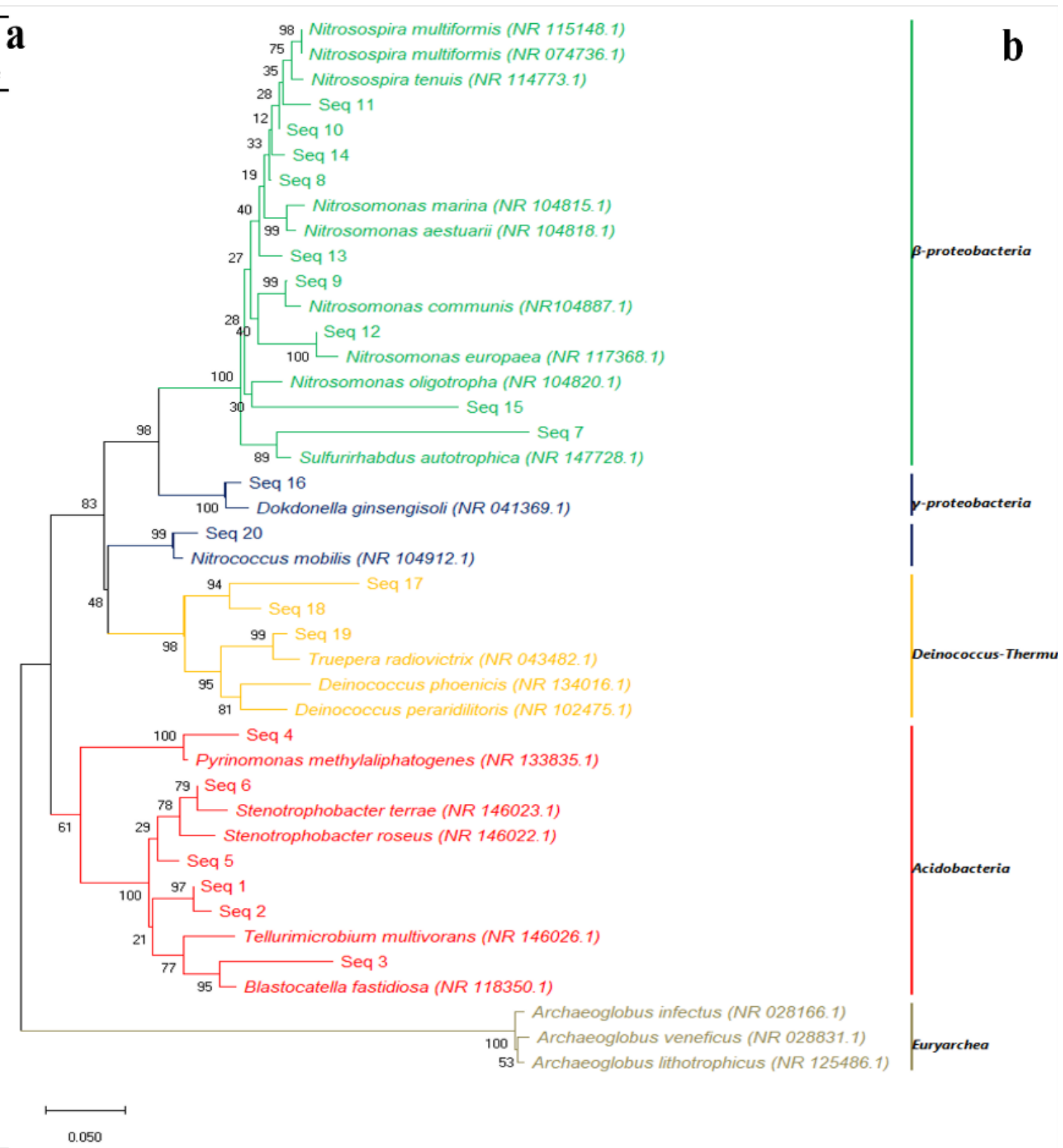

Figure Captions Supplementary material

Fig. S1 Heat map generated from intensities of DGGE gel band of the nitrifying sludge throughout the operating cycles with 60 mg 2-CP-C/L and different initial ammonium concentrations. The white color indicates absence or non-detection of the species and the black color indicates the highest proportion of individuals found. The sequenced bands are indicated in the figure (first column).

Fig. S2 a: identification of nucleotide sequences analyzed from DGGE bands of the nitrifying sludge throughout the cycles of operation with 60 mg of 2-CP-C/L and with different initial ammonium concentrations, using the base of the 16s rRNA of bacteria of the NCBI GenBank. b: Phylogenetic tree was generated based on the neighbor-joining method and the Jukes-Cantor substitution model of the 16S rRNA-DGGE sequences and related sequences from the NCBI GenBank bacterial 16s rRNA database. The bar represents five nucleotide substitutions per 100 nucleotides. The nodal robustness of the tree was assessed using 1000 bootstrap replicates. The NCBI GenBank accession number for each bacterium is shown in parentheses.
